# Copper microenvironments in the human body define patterns of copper adaptation in pathogenic bacteria

**DOI:** 10.1371/journal.ppat.1010617

**Published:** 2022-07-21

**Authors:** Francesca Focarelli, Andrea Giachino, Kevin John Waldron

**Affiliations:** Biosciences Institute, Faculty of Medical Sciences, Newcastle University, Newcastle upon Tyne, United Kingdom; Carnegie Mellon University, UNITED STATES

## Abstract

Copper is an essential micronutrient for most organisms that is required as a cofactor for crucial copper-dependent enzymes encoded by both prokaryotes and eukaryotes. Evidence accumulated over several decades has shown that copper plays important roles in the function of the mammalian immune system. Copper accumulates at sites of infection, including the gastrointestinal and respiratory tracts and in blood and urine, and its antibacterial toxicity is directly leveraged by phagocytic cells to kill pathogens. Copper-deficient animals are more susceptible to infection, whereas those fed copper-rich diets are more resistant. As a result, copper resistance genes are important virulence factors for bacterial pathogens, enabling them to detoxify the copper insult while maintaining copper supply to their essential cuproenzymes. Here, we describe the accumulated evidence for the varied roles of copper in the mammalian response to infections, demonstrating that this metal has numerous direct and indirect effects on immune function. We further illustrate the multifaceted response of pathogenic bacteria to the elevated copper concentrations that they experience when invading the host, describing both conserved and species-specific adaptations to copper toxicity. Together, these observations demonstrate the roles of copper at the host–pathogen interface and illustrate why bacterial copper detoxification systems can be viable targets for the future development of novel antibiotic drug development programs.

## Author summary

Copper is required by both animals and bacteria in small quantities as a micronutrient. During infection, the mammalian immune system increases the local concentration of copper, which gives rise to copper toxicity in the pathogen. In turn, bacterial pathogens possess specialized systems to resist this copper toxicity. Copper also plays important, indirect roles in the function of the immune system. In this review, we explain the diverse roles of copper in the human body with a focus on its functions within the immune system. We also describe how bacterial pathogens respond to the copper toxicity that they experience within the host during infection, illustrating both conserved copper homeostasis and detoxification systems in bacteria and species-specific adaptations that have been shown to be important to pathogenicity. The key role of copper at the host–pathogen interface and the essential requirement for pathogenic bacteria to resist copper toxicity makes the protein components that confer resistance on pathogens potential targets for future development of novel antibiotic drugs.

## Introduction

Copper is an essential micronutrient for both prokaryotes and eukaryotes. Its redox properties are exploited in biochemical reactions by copper-dependent enzymes. Therefore, during infection, invading microorganisms must acquire copper from the host to maintain copper supply to essential cuproenzymes. On the other hand, too much copper is toxic [1]. Copper’s high affinity for protein metal-binding sites (MBS) can result in the displacement of iron from enzyme catalytic sites, particularly iron-sulfur clusters [2]. Moreover, copper inhibits peptidoglycan cross-linking by *LD-*transpeptidases [3] and the maturation of lipoproteins in the gram-negative bacterial cell envelope [4], and induces protein aggregation by catalyzing the cross-linking of cysteine residues and the formation of non-native disulfide bonds [5]. Finally, copper participates in Fenton-like chemistry, leading to the production of reactive oxygen species and other toxic radicals [6]. Thus, both host and microorganism possess homeostatic systems that carefully regulate cellular copper bioavailability to prevent toxicity. Together, this presents an opportunity for the immune system to utilize elevated local copper concentrations as a biocidal weapon with which to attack invading pathogens [7,8].

Research studies have long supported a role for copper in the function of the immune system. It has been known for some time that copper deficiency impairs immune function [9,10]. Mammal models fed a copper-deficient diet are more prone to infection, and copper-rich diets increase their ability to fend off invading pathogens [11–15]. Copper deficiency in humans causes a number of symptoms, including neutropenia [16]. Recent evidence has begun to explain the reason for these observations mechanistically. Copper is used as a bactericidal agent within macrophages, accumulating in phagolysosomes during infection [8,17,18], and both systemic and localized copper concentrations in mammals are increased during infection [11,12].

The general principles of copper handling in bacteria and in mammals have been recently separately reviewed [1,19]. Here, we focus on the role of copper at the host–pathogen interface, summarizing the evidence for a crucial role for copper excess in host immunity to infection and illustrating the opposing role of copper detoxification systems encoded by human pathogens. We highlight the importance of copper microenvironments at different host/pathogen interfaces and show that pathogens colonizing the same niche adopt similar copper response strategies.

## Copper distribution in the mammal host

Copper is essential for all animal cells to supply crucial copper-dependent enzymes including cytochrome *c* oxidase and superoxide dismutase (SOD), among others. Both systemic and cellular mechanisms of copper homeostasis are needed to ensure adequate copper incorporation from the diet, its distribution to and within the tissues, its intracellular trafficking and regulation, and its supply and incorporation into individual cuproenzymes. However, the copper demand of different cell types within a multicellular organism is variable (Table 1). Red blood cells, for example, are devoid of cytochrome *c* oxidase, but still require copper for SOD. Copper is also required for the biosynthesis of ceruloplasmin and other serum copper-binding proteins, which are primarily secreted by hepatocytes. Ceruloplasmin is the primary copper carrier in mammalian blood, delivering copper from hepatocytes to tissues, whereas other plasma carriers such as albumin and the macroglobulin transcuprein bind the metal on initial entry into the circulation from intestinal cells [20]. Ceruloplasmin also possesses copper-dependent ferroxidase activity that plays a key role in iron transport and uptake [21].

**Table 1:** Copper content of human organs, tissues, and bodily fluids.

**Tissue/organ**	**Copper μg/g**	**Total mg copper for average 70-kg person**
Kidney	12 ± 7 (19)	3.2
Liver	6.2 ± 0.8 (9)	9.9
Brain	5.2 ± 1.1 (10)	8.8
Heart	4.8 ± 1.9 (14)	1.6
Skeleton	4.1 ± 1.3 (8)	45.5
GI tract	1.9 ± 0.9 (12)	2.8
Spleen	1.5 ± 0.4 (14)	0.2
Lungs	1.3 ± 0.4 (11)	1.3
Blood	1.1 ± 0.1 (5)	6.2
Muscle	0.9 ± 0.3 (7)	26.2
Skin	0.8 ± 0.4 (9)	3.8
Adipose	0.2, 0.3 (2)	3.0
Hair	2 ± 6 (21)	—
Nails	20 ± 17 (10)	—
**Fluid**	**Copper μg/ml**	**Copper μM**
Blood		
Whole	1.1 ± 0.13 (5)	17 ± 2
Plasma	1.13 ± 0.15 (70)	18 ± 2
Lymph	1.17 (1)	18
Sweat		
Men	0.6 (1)	9
Women	1.5 (1)	24
Saliva	0.22 ± 0.08 (4)	3 ± 1
Fallopian secretions	1.1 (1)	17
Seminal fluid	0.5, 1.5 (2)	8,24
Cerebrospinal fluid	5 ± 2 (4)	78 ± 31
Pleural fluid	0.60 (1)	9
Synovial fluid	0.2, 0.5 (2)	4, 8
Aqueous humor	0.14 (1)	2
Gastric juice	0.4 (1)	6
Bile	4.0 ± 1.9 (5)	63 ± 30
Gallbladder	(1.5 to 7.5)	(23 to 11)
Common bile duct	(0.3 to 10.5)	(5 to 160)
Urine	0.05 to 0.4^†^ (1)	1 to 6

Values are reproduced from [100]. Values are expressed as mean ± standard deviation when 3 or more studies were available, or as individual reports in the case of 2 or fewer studies (number of reports in parentheses), or as minimum–maximum ranges when the original study expressed them this way.

^†^Values for urine are μg/mg creatinine.

GI, gastrointestinal.

Copper uptake by mammalian cells utilizes a family of copper transporters, CTR, which are unique to eukaryotes and absent from bacterial genomes. Conversely, the proteins involved in intracellular copper trafficking and detoxification of excess copper are conserved across the tree of life: the human P-type ATPases that transport copper across membranes to remove it from the cytosol (ATP7A/B) are closely related to the copper efflux ATPases of bacteria and archaea (generally called CopA), and the soluble metallochaperones that assist their function in eukaryotic cells (ATOX1) have paralogues in many prokaryotes (generally CopZ).

**Fig 1:**
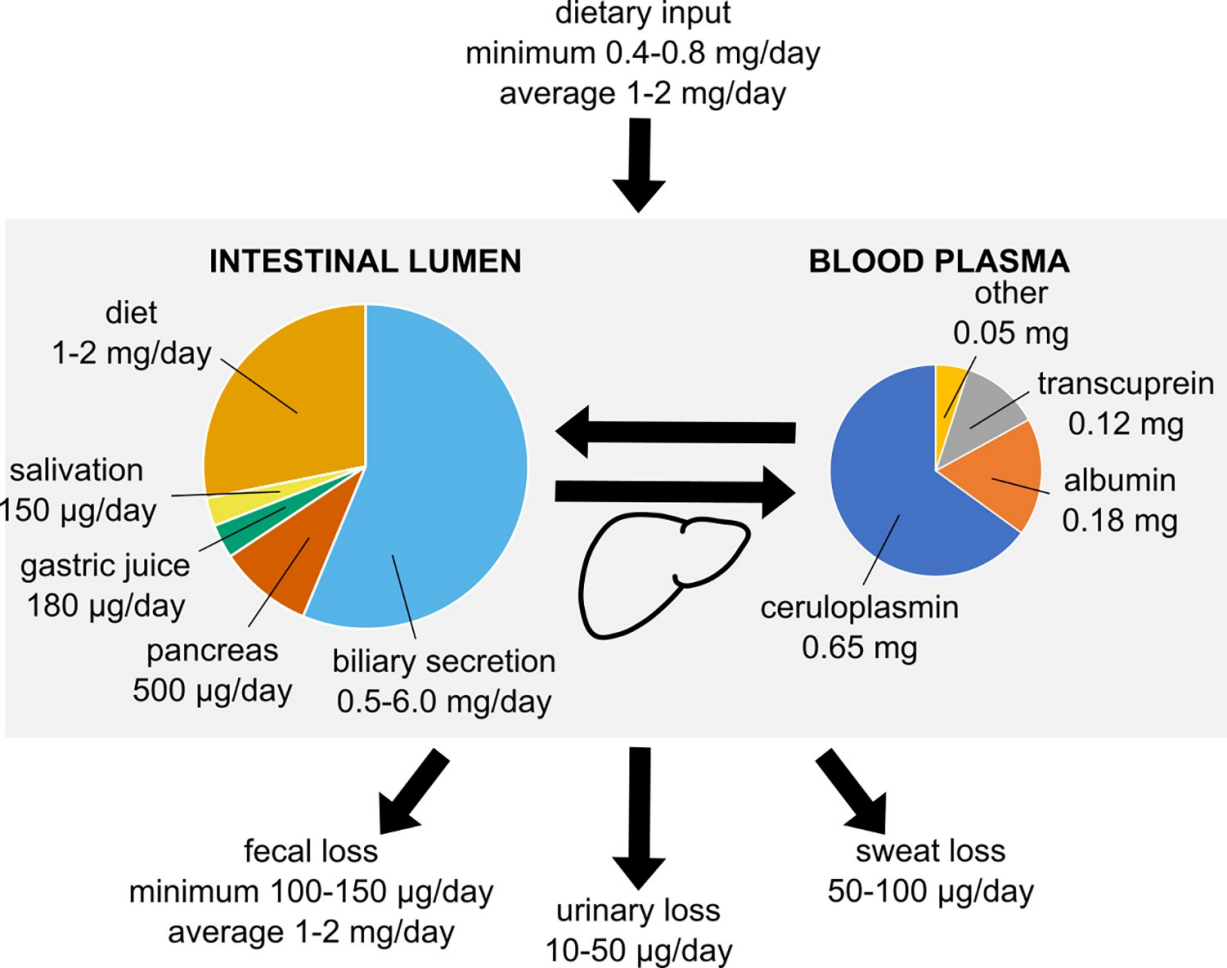
Copper homeostasis in the human body. Under copper homeostatic conditions, the amount of copper that enters an adult human body is the same that leaves the body. Because an average diet provides more copper than the minimum amount required (top), most dietary copper is excreted through feces (bottom). Importantly, most of the copper in the gastrointestinal tract is of endogenous origin (left) so that copper homeostasis is primarily maintained by balancing secretion and reabsorption, rather than through the diet. Circulating copper (right) is mostly bound to ceruloplasmin, and only 5% of circulating copper is not bound to carrier macromolecules. The majority of the copper secreted into the digestive tract (through bile) or into the bloodstream (through ceruloplasmin) comes from the liver, which is the primary organ regulating copper homeostasis.

Copper homeostasis in mammals is tightly regulated at the organism level (Fig 1, Table 1). The average human body contains about 110 mg copper [22], with only minimal amounts of copper exchanged with the environment. Circulating copper is lost through urine (10 to 50 μg/day), sweat (50 to 100 μg/day), and feces (100 to 150 μg/day) [23,24]. The minimum dietary copper required to compensate these losses and maintain homeostasis is 0.4 to 0.8 mg/day [25]. Complete turnover of bodily copper takes approximately 12 days [26].

A lot more copper is secreted into the gastrointestinal tract and subsequently reabsorbed in the small intestine. This comes mostly from bile (between 0.5 and 6 mg/day depending on estimates), but also saliva (approximately 150 μg/day), gastric (180 μg/day), and pancreatic juices (500 μg/day) [27] (Table 1). Dietary copper contributes an additional 1 to 2 mg/day, which is more than the minimum amount required for homeostasis [24]. Excess copper is not absorbed, so that a total 1 to 2 mg copper leaves the body through feces daily [26].

Importantly, much copper in the human gastrointestinal tract is of endogenous origin (1.6 to 7 mg/day) as opposed to dietary (1 to 2 mg/day) [27]. Copper homeostasis in the body is maintained by adjusting the rates of biliary secretion and dietary absorption, placing the intestine and the liver at the center of mammalian copper homeostasis [20,26].

Most of the copper contained in the human body is bound to cuproproteins and enzymes, and the amount of circulating copper is low. Whole blood contains approximately 6 mg copper [20], but only 1 mg is in plasma (Table 1). Moreover, 65% of plasma copper is tightly bound to ceruloplasmin, followed by 18% in albumin and 12% in transcuprein [22]. The remaining 5% of plasma copper is bound to peptides and amino acids, including glutathione.

Upon uptake by the intestine and subsequent secretion into the blood, circulating copper is quickly absorbed by the liver and then slowly secreted in ceruloplasmin-bound form [20,21]. Ceruloplasmin is an abundant multicopper oxidase in serum, which mediates iron homeostasis by oxidizing Fe(II) into Fe(III) for incorporation into ferritin [21,28] and constitutes an important source of copper in plasma [29] and urine [11,13]. Ceruloplasmin production by the liver increases during infection [21], causing an increase in copper circulating throughout the body [30]. A similar cycle of uptake and release also occurs in the kidney, ensuring efficient buffering of copper levels in the blood.

**Fig 2:**
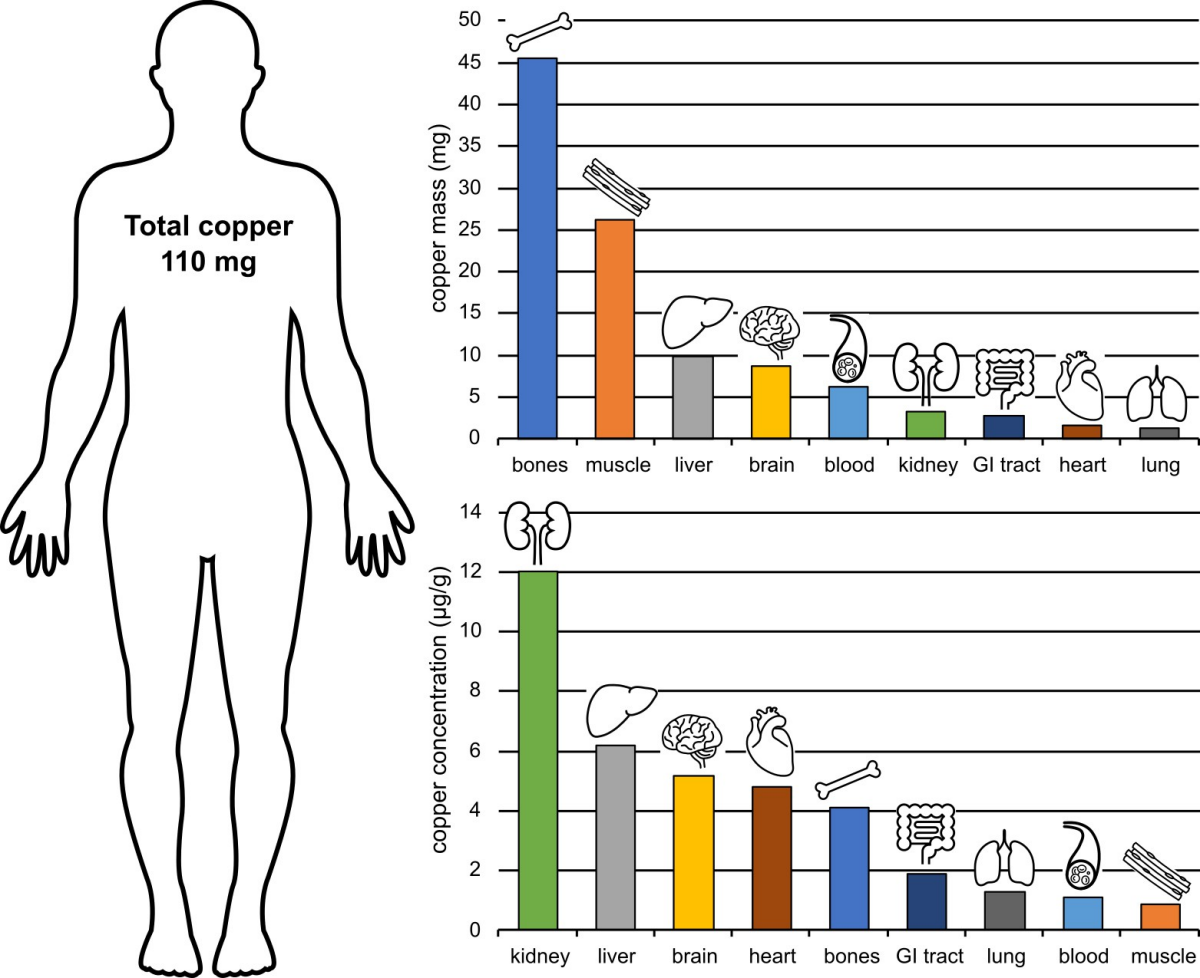
Copper distribution across organs. An average adult human contains approximately 110 mg of copper, the majority of which is ligated in the bones (including bone marrow) and skeletal muscle (top). However, the highest concentration of copper is found in the kidney and liver (bottom), which are the organs chiefly responsible for copper homeostasis. Notice that common infection sites—lung, blood, and the GI tract—can be considered copper poor. GI, gastrointestinal.

The highest concentration of copper is found in the liver (approximately 6.2 μg/g weight or approximately 10 mg total), followed by brain (5.2 μg/g, 8.8 mg total) and heart (4.8 μg/g) (Fig 2). Significant copper is also found in the kidney, varying between 4 and 12 μg/g. Quantitatively, most copper is found in the bones and bone marrow (46 mg) and skeletal muscle (26 mg) (Fig 2), although at a lower concentration than in other tissues (0.9 μg/g in skeletal muscle) [20,22,27].

## Copper in systemic infection

Copper resistance contributes to tissue colonization and inflammation for many pathogens, including uropathogenic *Escherichia coli* [31], *Staphylococcus aureus* [13], *Streptococcus pneumoniae* [32], *Mycobacterium tuberculosis* [12,33], *Listeria monocytogenes* [34], *Neisseria gonorrhoeae* [35], *Klebsiella pneumoniae* [36], and *Pseudomonas aeruginosa* [37]. Therefore, all these pathogens are thought to encounter significant concentrations of toxic copper at least in some phases of their pathogenesis.

Copper toxicity at the host–pathogen interface can occur in 2 ways: either the pathogen is exposed to copper in the extracellular space, such as on the surface of an epithelium, or intracellularly, after being engulfed by phagocytes. An increase in circulating copper during infection, and other pathological conditions, has been recognized for more than 60 years [23,38], such that copper accumulates at high levels in the blood and peripheral tissues during systemic infection [38]. This increase is driven by the acute phase overexpression and secretion of ceruloplasmin (Cp) from the liver, which is induced by pro-inflammatory agents such as interferon gamma (IFNγ), interleukin (IL) 1-β, and bacterial lipopolysaccharide (LPS) [39–41]. Epithelial cells also overexpress ceruloplasmin during infection, driving its accumulation in airways [40] and the urinary tract [11]. Acute phase ceruloplasmin overexpression upon inflammation is thought to mediate increased copper availability to other cytotypes to support the cell-mediated response to invading organisms [11,13]. In addition, increased extracellular copper also contributes to pathogen clearance through phagocyte-independent mechanisms, whose precise molecular mechanisms are currently under investigation [17,42].

Another copper-binding protein that is expressed during inflammation is the pro-inflammatory mediator calgranulin C (S100A12, also known as EN-RAGE) [43], which is produced by granulocytes and mediates the activation of endothelium, mononuclear phagocytes, and lymphocytes [43]. It has been suggested that the copper-S100A12 complex might sequester copper as part of the nutritional immunity response, similarly to how calprotectin (S100A8/S100A9, also known as the cystic fibrosis antigen, calgranulin A/B, and MRP-8/9) sequesters metals [44]. However, these interesting possibilities currently await experimental confirmation.

Importantly, the need for copper tolerance varies across different infection sites. *S*. *pneumoniae* encounters copper toxicity in the nasopharynx and lungs, but not in blood [32], and *M*. *tuberculosis* strains impaired in copper tolerance are less virulent in the lung, but not the spleen [12]. Copper tolerance plays a role in *Salmonella* Typhimurium colonization of liver and spleen, but not of lymph nodes [42]. Moreover, copper abundance and the presence of other bactericidal agents also varies across infection sites. Consequently, pathogens colonizing different environments have evolved distinct mechanisms to deal with copper toxicity.

## Copper in the immune system

**Fig 3:**
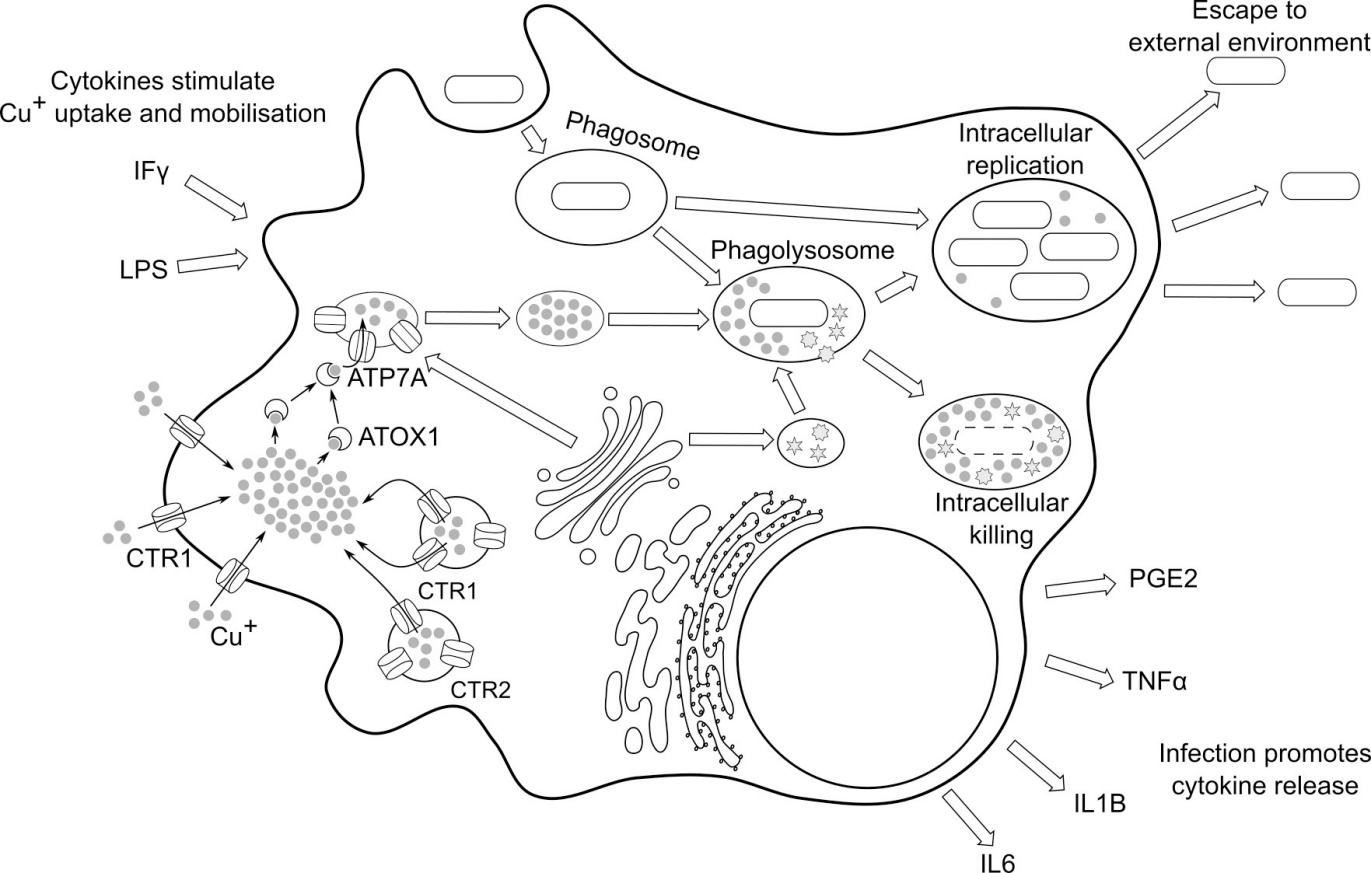
Copper delivery to the phagolysosome. Pro-inflammatory signals, such as IFγ and LPS, stimulate professional phagocytes to overexpress the copper-transport proteins CTR1, CTR2, and ATP7A. Copper concentration (gray circles) in the phagocyte cytoplasm increases through copper import by CTR1, and copper mobilization from intracellular storage vacuoles by CTR1 and CTR2. Cytosolic copper is captured by the ATOX1 chaperone and delivered to ATP7A, which is trafficked to the early lysosome from the Golgi apparatus and drives copper accumulation in the early lysosome. Upon phagocytosis of an invading microorganism, the maturing phagosome fuses with copper-rich lysosomes, exposing the microorganism to high copper levels. If copper fails to accumulate in the phagolysosome, lysis of engulfed bacteria is compromised and intracellular proliferation can occur. Copper is also required for the biosynthesis of many extracellular signals, including PGE2, TNFα, and IL1B and IL6. IFγ, interferon-γ; IL, interleukin; LPS, lipopolysaccharide; PGE2, prostaglandin E2; TNFα, tumor necrosis factor α.

The role of copper in the immune system is manifold. As well as a direct antibacterial role in macrophage killing [8,17,18] (Fig 3), copper also plays a role in the respiratory burst, the early production of reactive oxygen species in the phagolysosome [14]. Copper deficiency reduces the production of IL-2 by T-lymphocytes and mononuclear cells by inhibiting transcription of the IL-2 gene [45] and repressing T-cell maturation and proliferation [10]. Secretion of other inflammatory mediators, including tumor necrosis factor alpha (TNF-α), IL-1β, IL-6, and prostaglandin E(2) (PGE2) is also reduced in copper-deprived promonocytic cells [46] (Fig 3).

The mature phagolysosome of phagocytic cells is a copper-rich environment, and high copper levels within the phagolysosome contribute to bacterial clearance after phagocytosis [7,8]. Copper accumulation in the phagolysosome is a tightly regulated process mediated by copper transporters and induced by pro-inflammatory agents such as IFNγ and bacterial LPS [8,17,18] (Fig 3).

In response to pro-inflammatory agents, macrophages upregulate the expression of the copper transport protein, CTR1 [17], which localizes at the cell membrane and mediates copper uptake from the extracellular milieu [47]. CTR1 also localizes to intracellular vesicles, where it contributes to mobilizing intracellular copper storage in conjunction with the regulatory CTR2 [48]. Pro-inflammatory agents also increase the expression of CTR2 [17], which localizes to intracellular vesicles and induces the proteolytic cleavage of vesicle-bound CTR1 into a truncated form (tCTR1) [49] (Fig 3). tCTR1 then exports copper from vesicular storage into the cytoplasm [50].

Taken as a whole, inflammation-induced expression of CTR1 and CTR2 increases copper availability in the cytosol by a combination of increased copper uptake from the environment, and copper mobilization from endosomal compartments [17,49,50]. Copper delivery from the cytosol to the phagolysosome occurs through the copper-transporting ATPase alpha (ATP7A) in conjunction with the intracellular chaperone ATOX1, which chelates copper in the cytosol and delivers it to ATP7A [51]. Under non-inflammatory conditions, ATP7A localizes in the trans-Golgi, where it contributes to copper delivery to maturing cuproproteins [52]. Pro-inflammatory agents induce overexpression of both ATOX1 and ATP7A [17] and stimulate ATP7A trafficking to maturing lysosomes [8] (Fig 3). ATP7A-containing lysosomes accumulate copper [17] prior to their merging with the phagosome, ultimately resulting in copper delivery to the phagolysosome [18].

Copper delivery to the phagolysosome requires adequate extracellular levels of copper, as well as the activity of CTR1, CTR2, and ATP7A. If the process is impaired, through either extracellular copper chelation [17,46] or silencing of ATP7A [7,8], it results in impaired phagosomal killing of invading bacteria. Conversely, priming of macrophages with copper increases their ability to destroy engulfed bacteria [8].

## Copper-mediated pathogen killing in the phagolysosome

The mature phagolysosome is a copper-rich environment, accumulating copper in the range of tens to hundreds of micromolar [18]. Copper accumulation is mediated by the overexpression of ATP7A and its vesicular delivery from the trans-Golgi to the surface of maturing phagolysosomes [7,8]. Copper enters engulfed bacteria [53] and contributes to phagocytic killing through a variety of mechanisms, which have been extensively reviewed elsewhere [1]. In response to phagosomal copper overload, engulfed bacteria upregulate copper-homeostasis genes, such as that encoding the copper-efflux pump CopA [54].

Bacterial resistance to copper toxicity is a key virulence determinant, and it contributes to bacterial survival within macrophages. Bacterial strains lacking copper-tolerance genes are more sensitive to phagosomal killing, as demonstrated in *E*. *coli* [8], *S. enterica* serovar Typhimurium [7,53], *S*. *pneumoniae* [55], and *S*. *aureus* [56,57]. Conversely, in pathogenic strains that possess mobile genetic elements (MGE) encoding for copper hypertolerance, these MGE further enhance intraphagosomal survival [56,57]. Other intracellular pathogens also require copper homeostasis to survive within host cells, as is the case for *N*. *gonorrhoeae* within cervical epithelial cells [35].

The mechanisms of copper-mediated killing within phagocytes are multifaceted. Following engulfment, the phagosome matures into a bactericidal environment by the regulated fusion of vesicles, particularly lysosomes and proteases, progressive pH acidification (to final pH 4.5) and nutrient deprivation [58]. Eventually, the mature phagolysosome contains high concentrations of copper and zinc [18,59,60], low concentrations of iron [61], radical-inducing species such as reactive oxygen, nitrogen, and chlorine species [58,62,63], and other bactericidal proteins such as lysozyme, defensins, phospholipases, and proteases [58]. Copper ions participate in Fenton-like chemistry with ROS and RNS, amplifying the generation of reactive radicals [6]. Moreover, both copper and zinc can mismetalate ferroenzymes [2,64]; as a consequence, copper and zinc toxicity further enhance the effects of iron removal from the phagolysosome [61] and inflamed tissues [65] as part of nutritional immunity [59].

Interestingly, several pathogens display elegant mechanisms to escape the macrophage intracellular killing. For example, pathogenic *S*. Typhimurium inhibits the delivery of NADPH oxidase and inducible nitric oxide synthase (iNOS) to the maturing phagosome, preventing both the early oxidative burst [66] and long-term RNS toxicity [67]. In addition, this bacterium counteracts high copper concentrations in the phagolysosome by successfully preventing copper entry into the bacterial cell [59]. Similarly, *M*. *tuberculosis* also inhibits iNOS recruitment to the phagolysosome [68] and also interferes with vesicle fusion with the phagosomal compartment, ultimately resulting in the creation of a vacuole that is permissive for bacterial growth [69]. In other words, pathogens can avoid phagosomal toxicity in two different ways: either by preventing the maturation of the bactericidal compartment or by coping with bactericidal factors after phagolysosomal maturation.

## Bacterial adaptations to host copper flux

In the past decade, the role of metal ions at the host-microbe interface has attracted increasing attention. Copper toxicity, in particular, is an important emerging field due to copper’s powerful antimicrobial properties, especially in the age of rapidly spreading antibiotic resistance.

Yet, despite recent advances, the role of copper at the host–pathogen interface is far from fully understood. One challenge in investigating copper homeostasis stems from the key differences in host microenvironments that we discussed earlier in this work: copper distribution in the human body is not uniform, and pathogens can be exposed to significant fluctuations in copper concentration both in space (different bodily niches) and time (e.g., as a consequence of immune system activation). Furthermore, copper tolerance in bacteria is not limited to the physical handling of copper, but also includes mechanisms to adapt microbial metabolism to repair, or bypass, the toxic effects of copper [1].

**Fig 4:**
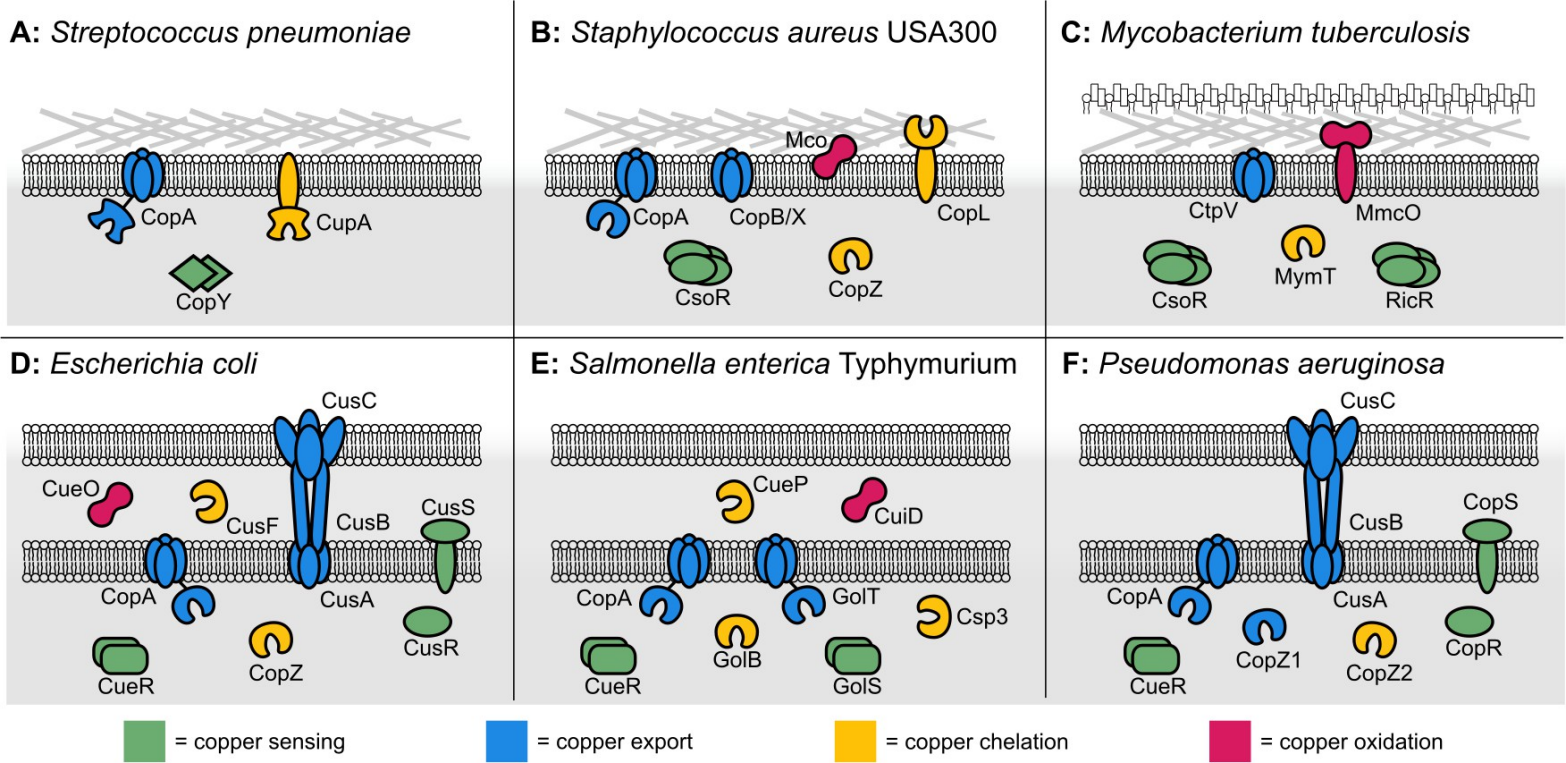
Copper resistance in human pathogens. Despite individual variations, most human pathogens share a common pattern of copper resistance proteins, involving four key functions: copper sensing (green), export (blue), chelation (ochre), and oxidation (magenta). The first three functions (sensing, export, and chelation) are common to the majority of pathogens, and many also include the fourth function (oxidation). In some cases, the copper-homeostasis suite can also vary across different strains or serotypes (most notably in *S*. *aureus* and *S*. *enterica*). Copper oxidation only occurs outside of the cytosol, and it may contribute to slowing down copper entry into the cytosol. All P-type ATPases belong to a single family (CopA) which is conserved across the tree of life (including eukaryotes). Different subfamilies of the ATPase may feature different terminal metal-binding domains, and these are homologous to that organism’s cytosolic chaperone. **(A)** *Streptococcus pneumoniae* contains a minimal set of copper-homeostasis proteins, including the CopY sensor, the CopA exporter, and the CupA chaperone. **(B)** The common USA300 strain of *Staphylococcus aureus* contains two copper-efflux pumps (CopA and CopB/X) as well as a cell-surface associated chelator (CopL) and multicopper oxidase (Mco). **(C)** *Mycobacterium tuberculosis* has a minimum copper-homeostasis system with the CtpV efflux pump, the MymT metallothionein, and the MmcO oxidase; however, this system is controlled by two separate (but homologous) regulators: CsoR for CtpV, and RicR for MymT and MmcO. **(D)** As a gram-negative organism, *Escherichia coli* features a rich suite of periplasmic copper-homeostasis proteins, including the CusSR two-component sensory system, the CusCBA efflux pump, the CusF chaperone, and the CueO oxidase. Copper homeostasis in the *E*. *coli* cytoplasm is minimal, with only the CueR regulator, the CopA pump, and the CopZ chaperone dealing with copper homeostasis in this compartment. **(E)** Although related to *E*. *coli*, *Salmonella enterica* sv. Typhimurium lacks systems for periplasmic copper sensing or efflux; conversely, it carries a duplication of the cytoplasmic copper-homeostasis system (the Gol proteins) and an additional copper-storage protein (Csp3). **(F)** *Pseudomonas aeruginosa* features copper sensing and efflux both in the cytosol and the periplasm, but no periplasmic copper chelator or oxidase have been identified to date.

Yet, our increasing understanding of the bacterial copper response has highlighted some important similarities across human pathogens, as well as some key differences (Fig 4). In the following sections, we compare the copper response of six important human pathogens (*S*. *aureus*, *S*. *pneumoniae*, *M*. *tuberculosis*, uropathogenic *E*. *coli*, *S. enterica* serovar Typhimurium, and *P*. *aeruginosa*) to highlight its key role in the host–pathogen interaction.

Importantly, this group of pathogens includes diverse organisms from the gram-positive, gram-negative, and mycobacterial groups (Fig 4). Some of these organisms are closely related (*E*. *coli* and *S*. *enterica*) while others are not. Finally, these pathogens colonize different niches in the body, yet some of them can occupy the same or similar niches: for example, both *S*. Typhimurium and *M*. *tuberculosis* are intracellular pathogens, while *S*. *pneumoniae* and *M*. *tuberculosis* both occupy the human respiratory tract. By linking these pathogens’ copper response with their distinctive lifestyle, we show how different bacterial adaptations can be understood in terms of their respective adaptation to different host niches.

Crucially, the suite of copper resistance proteins in human pathogens is remarkably similar, despite individual variations (Fig 4). Three core functions of the copper response are universally conserved across pathogens: copper sensing, copper export, and copper chelation. A fourth mechanism, copper oxidation, is also present in many but not all organisms.

## Conserved mechanisms of copper sensing

Firstly, all bacteria that are exposed to copper stress need a way to sense it. Therefore, copper-sensing systems are nearly universally present across pathogens. These systems belong to ancient protein families, and similar gene homologues are common to multiple taxa [70]. For example, gram-negative bacteria (particularly proteobacteria) often sense cytosolic copper through a MerR-like sensor, whose prototypical member is the CueR sensor from *E*. *coli* [71]. A CueR homologue is also present in *P*. *aeruginosa* and *S*. Typhimurium, and the latter carries two homologues of this protein with distinct but related functions in copper tolerance [72].

By contrast, monodermic bacteria (and mycobacteria) usually sense cytosolic copper via 1 of two transcriptional repressors: the four-helix-bundle CsoR family or the helix-turn-helix CopY family [70]. In the case of *M*. *tuberculosis*, the gene for the CsoR-like regulator is duplicated, and a second homologue (RicR) is responsible for fine-tuning the copper response [33]. Therefore, even though all bacteria require a way to sense and respond to cytosolic copper overload, the precise mechanisms by which this is accomplished may differ.

Another important difference in copper sensing occurs in didermic (gram-negative) bacteria. Because gram-negative cells include a periplasmic space, they also need to maintain copper homeostasis in this compartment. Consistently, many copper-homeostasis proteins can be found in this compartment. However, only some gram-negatives also encode a sensor for periplasmic copper: the two-component CopSR system (also known as CusSR in *E*. *coli*) [70]. Importantly, *S*. Typhimurium lacks a CopSR homologue but encodes a complex suite of periplasmic copper response proteins [53], suggesting that this bacterium has evolved to mount a cell-wide copper response instead of differentiating between different compartments.

Crucially, extracytosolic copper homeostasis is not restricted to gram-negative bacteria. Both *S*. *aureus* USA300 and *M*. *tuberculosis* encode one or more surface-associated copper-homeostasis proteins, which contribute to detoxifying copper before it enters the cell [56,73]. It is significant that the CopSR system is also found in some gram-positive organisms, including *Corynebacterium glutamicum* [74]. This further supports the model of the cell envelope as a key player in copper homeostasis, even in the absence of a periplasmic space.

Overall, the patterns of copper response regulation identify three different classes of pathogens: (a) pathogens that only encode one copper sensor, including *S*. *pneumoniae* and *S*. *aureus*; (b) pathogens that differentiate the copper response based on subcellular compartment, such as *E*. *coli* and *P*. *aeruginosa*; and (c) pathogens that fine-tune the copper response depending on the presence of additional toxicants and/or via multiple cytosolic copper sensors, such as *M*. *tuberculosis* and *S*. Typhimurium.

## Mucosa-associated pathogens experience a stable copper environment

Importantly, these patterns of copper regulation strongly correlate with the pathogen’s lifestyle, likely reflecting the unique distribution of copper in those compartments. *S*. *pneumoniae* and *S*. *aureus* are mucosal colonizers, which thrive in association with epithelia and frequently form epithelium-associated biofilms. In this scenario, the copper concentration encountered by these pathogens in the primary environment is relatively constant in time and space. Therefore, a single copper sensor is sufficient to detect (and respond to) copper abundance in the pathogen’s environment.

Evidence of a constant copper pressure on mucosal epithelium-colonizing pathogens is also provided by the expansion of the copper suite in pathogenic *S*. *aureus*, which (as a clade) includes different strains adapted to varying copper levels in the host.

In the case of *S*. *aureus*, only the “minimal” suite of one sensor (CsoR), one efflux pump (CopA), and one copper chaperone (CopZ), is shared by the entire clade. However, methicillin-resistant USA300 isolates carry additional loci for copper tolerance on MGEs, the same that encode antibiotic resistance in these strains [56,57,75]. These supplementary loci usually carry genes for the surface-exposed chelator CopL [56,73], and a second P-type ATPase (denoted CopX or CopB, depending on the isolate). In some cases (but not always), these are also accompanied by the Mco multicopper oxidase.

Multiple lines of evidence suggest that the additional copper-homeostasis genes in USA300 are important for virulence. All three additional genes mediate copper hypertolerance [56,57,73], and this hypertolerance can be transferred horizontally to copper-sensitive strains [57]. They are also upregulated upon phagocytosis and contribute to the enhanced survival of USA300 within macrophages [56,57]. Finally, the acquisition of *copB*/*X* occurred independently in different strains, suggesting that these strains experience a common evolutionary pressure to select for additional copper-homeostasis mechanisms [75].

Importantly, the acquisition of additional copper response proteins was not accompanied by the development of a separate copper sensor, since the additional genes are all CsoR-regulated [57]. This is consistent with the fact that USA300 colonize the same body niches as the methicillin-sensitive parent; in other words, the additional genes carried by USA300 enhance the ability of *S*. *aureus* to colonize its native niche, instead of mediating a different lifestyle. Because copper inhibits the formation of *S*. *aureus* biofilms [76], it is likely that increased copper tolerance in USA300 is important for its increased ability to colonize the host.

## Copper homeostasis in the gram-negative periplasm reflects copper levels in different biological niches

By contrast, gram-negative bacteria such as *E*. *coli* and *P*. *aeruginosa* possess an additional subcellular compartment, the periplasm, which features different copper chemistry compared to the cytoplasm. Both *E*. *coli* and *P*. *aeruginosa* possess a 2-component sensory system for detecting periplasmic Cu(I) (CusSR and CopSR, respectively) in addition to the cytosolic sensor (CueR) [77,78]. However, the functions of these systems in the context of cellular metal homeostasis are radically different and likely reflect the different niches colonized by these bacteria.

In *E*. *coli*, the CusSR system induces copper efflux from the periplasm when Cu(I) availability reaches micromolar levels [79] and is thought to constitute an adaptation to increased Cu(I) availability during anaerobic conditions, or to sudden increases in extracellular copper [79]. Importantly, mammalian gut-associated *E*. *coli* experiences drastic fluctuations in oxygen availability, changing between meals [80], along the intestinal tract [81] and from the tissue interface to the lumen [82]. Importantly, oxygen levels also influence copper availability by modulating the redox equilibrium between Cu(I) and Cu(II): anaerobic conditions increase Cu(I) availability [83], resulting in in increased copper influx and accumulation in *E*. *coli* [79]. As a consequence, the *E*. *coli* copper response is strongly induced during the transition from aerobic to anaerobic growth [84]. Taken together, the available evidence suggests that periplasmic copper homeostasis in *E*. *coli* evolved specifically to deal with copper fluctuations in the primary environment, as a consequence of variable oxygen levels.

By contrast, periplasmic copper homeostasis in *P*. *aeruginosa* is believed to be more tightly controlled [85]. The *P*. *aeruginosa* periplasmic two-component system CopSR reacts to subfemtomolar periplasmic Cu(I) and is thought to maintain the *P*. *aeruginosa* periplasm devoid of free copper [78]. At the same time, *P*. *aeruginosa* is one of the few bacteria for which a periplasmic copper-import route has been identified [86] and has been shown to scavenge copper from plasma during infection [87]. Indeed, copper tolerance (and likely acquisition) plays an important role in the establishment of preinfection communities of *P*. *aeruginosa* [88] and strains defective in copper homeostasis show decreased pathogenicity [37]. One interesting model that has been recently proposed is that *P*. *aeruginosa* is subjected to copper excess and starvation outside of the human body, and that adaptation to copper-related evolutionary pressure increases this bacterium’s ability to adapt to copper fluctuations within the human host [88].

Another similarity between *E*. *coli* and *P*. *aeruginosa* is the secretion of copper-binding molecules that sequester copper outside of the cell. In *P*. *aeruginosa*, the secreted siderophores pyoverdine and pyochelin chelate copper outside the cell and increase cellular tolerance to toxic copper [89]. Similarly, many uropathogenic *E*. *coli* strains encode the siderophore yersiniabactin as part of the *Yersinia* high-pathogenicity island, whose expression is strongly induced during urinary tract infection [90]. Similarly to pyoverdine and pyochelin, yersiniabactin sequesters copper in the extracellular milieu, protecting uropathogenic *E*. *coli* from copper toxicity during infection [31]. At the same time, yersiniabactin also protects *E*. *coli* from the early respiratory burst in phagocytes [91] and is involved in copper acquisition during copper-limiting conditions [92], supporting the notion that successful pathogens such as *P*. *aeruginosa* and UPEC need both copper sequestration and acquisition to deal with varying copper levels within the host.

Crucially, another gram-negative bacterium that is evolutionary close to *E*. *coli*, *S*. *enterica*, does not possess a periplasmic copper sensor. Instead, periplasmic copper homeostasis in *S*. *enterica* is under the control of the cytosolic copper sensor, CueR [53]. This suggests that *S*. *enterica* has a lower requirement for carefully managed periplasmic copper, but instead requires increased protection of the cytosol, as evidenced by the presence of two cytosolic efflux pumps in *S*. *enterica* sv Typhimurium [53]. This is likely linked to the different lifestyles of *S*. *enterica*, a facultative intracellular pathogen, as opposed to *E*. *coli* and *P*. *aeruginosa*, whose intracellular phase plays a less-relevant role in pathogenesis [93].

## Intracellular pathogens recognize copper in conjunction with other phagolysosomal antimicrobials

Finally, the third class of pathogens includes the intracellular pathogens *S*. Typhimurium and *M*. *tuberculosis*, both of which have evolved to survive and replicate within phagocytic cells. As described earlier, the mammalian phagolysosome is a complex compartment, characterized by toxic copper levels in conjunction with a suite of other antimicrobial effectors. For intracellular pathogens, copper concentration comes as an all-or-nothing: they are either in a copper-poor environment (outside the macrophage or in a pathogen-adapted vacuole) or in a deadly, copper-rich environment (a maturing phagolysosome) [53]. Importantly, copper toxicity in the phagolysosome is accompanied by a suite of other antimicrobial factors, some of which feature potent synergistic effects with copper.

It is not surprising, therefore, that intracellular pathogens would have adapted to respond to toxic copper at the same time as they are also dealing with other antimicrobial factors. Even though they are not related, both organisms evolved a duplication of the “core” copper-sensing system (CueR in *S*. Typhimurium, CsoR in *M*. *tuberculosis*) generating a homologous but independent sensor (GolS in *S*. Typhimurium and RicR in *M*. *tuberculosis*). In both organisms, the primary copper sensor responds only to copper concentration [53,94], while the additional sensor responds to copper in conjunction with other cellular stressors: the *S*. Typhimurium GolS responds to copper and acid stress [72], while the *M*. *tuberculosis* RicR is activated by copper, cobalt, nickel and zinc, as well as reactive oxygen and nitrogen species [94]. Copper and nitric oxide also coregulate the expression of the Rip1/PdtaSR regulon in *M*. *tuberculosis*, which mediates a coordinated response to both stressors [95], although the exact mechanisms remain to be clarified. Importantly, all of these stressors are encountered alongside copper within macrophages [58].

The duplication of the copper response system in *S*. Typhimurium and *M*. *tuberculosis* is likely a convergent adaptation to intraphagosomal survival. We hypothesize that by acquiring a second copy of the respective copper sensor, both organisms have evolved the ability to distinguish between copper toxicity outside and inside the phagolysosome. Importantly, this explains the “atypical” copper homeostasis in *S*. Typhimurium, which differs significantly from closely related organisms [53]. In fact, copper homeostasis in *S*. Typhimurium is more similar to *M*. *tuberculosis* than *E*. *coli*, reflecting the sharing of a similar niche with the former, but not the latter. We propose that the co-occurrence of elevated copper and other antimicrobials in the phagolysosome caused the convergent evolution of an additional copper-detoxification system in *M*. *tuberculosis* and *S*. Typhimurium, which specifically responds to copper and other phagolysosome-associated stress factors.

## Conclusions

The accumulated evidence strongly indicates diverse and important roles of copper in immune function. Some roles for copper are direct, for example, the antibacterial accumulation of copper inside the phagosome of phagocytic cells, whereas other roles are indirect, such as copper-mediated effects on endocrine signaling that modulate the immune response. In contrast to other metals, such as manganese and iron, whose abundance is restricted by the host, pathogens seem to experience elevated copper concentrations during infection. Bacterial proteins that function to detoxify copper insult are known virulence factors in several globally important human pathogens. These prokaryotic components thus have potential as targets for small-molecule inhibitors as a route to the development of novel antibiotic therapeutics.

Several pathogens are observed to have undergone gene duplication events, increasing their copy number of key copper homeostasis genes. Where these duplication events were very ancient, such as in *S*. Typhimurium or *M*. *tuberculosis*, the duplicated genes have subsequently diverged to confer new functions such as to give fine control over the copper response to enable the bacterium to better survive inside host cells. But in the case of current epidemic lineages of *S*. *aureus*, the observed duplication was much more recent, confers hyperresistance to copper, and provides a selective benefit during interaction with immune cells. This raises the prospect that contemporary pathogen evolution is selecting for more copper-resistant phenotypes, which may have anthropogenic causes and thus be an increasing selection pressure.

At the same time, metal hyperresistance has been linked to the spread of resistance to other antimicrobials, crucially antibiotics [96,97]. One way in which this could occur is the coselection of metal and antibiotic resistance genes on the same MGEs. Another mechanism is cross-resistance, in which a metal resistance determinant (e.g., a multidrug efflux pump or a particular composition of the cell envelope) is also capable of mediating antibiotic resistance, even in the absence of dedicated antibiotic resistance genes [98]. At the same time, metal resistance determinants can also increase a bacterium’s sensitivity to certain antibiotics [99]. Clearly, the potential interactions of copper and other antimicrobials are multifaceted and constitute an area of active research. If copper is to be exploited as an antimicrobial in future, monitoring of the emergence and spread of such hyperresistant phenotypes among pathogens will be essential.
